# Tissue Data Explorer: a website template for presenting tissue sample research findings

**DOI:** 10.21105/joss.09218

**Published:** 2026-03-23

**Authors:** James A. Labyer, Erik Ferlanti, Martha Campbell-Thompson, Clayton E. Mathews, Wei-Jun Qian, James P. Carson

**Affiliations:** 1 Texas Advanced Computing Center, The University of Texas at Austin, Austin, TX; 2 Department of Pathology, Immunology and Laboratory Medicine, University of Florida, Gainesville, FL; 3 Biological Sciences Division, Pacific Northwest National Laboratory, Richland, WA

## Summary

The Tissue Data Explorer is a Python code repository that can be used to deploy a website for displaying 2D and 3D biomolecular datasets that have been collected from tissue samples taken from organs. The Tissue Data Explorer includes interfaces for loading and displaying image stacks, spatial maps of measurements taken from different areas within the tissue sample, 3D models of the tissue sample, and links to custom reports. Life sciences researchers can deploy an instance of the website as is, or they can fork the repository and modify the interfaces to accommodate additional data types. To enable easier customization, the website is written using the Plotly Dash visualization framework (Parmer et al., 2024). Dash allows users to build visuals using Python, which is likely to be easier for many biologists because Python knowledge is common among life sciences researchers (Mariano et al., 2020) (Boudreau, 2021). This is in contrast with other common web languages like JavaScript, which is popular among software developers (“Stack Overflow Developer Survey,” 2024) but has not achieved the same level of adoption with life sciences researchers (Sorani, 2012).

## Statement of Need

This software emerged from a project that aimed to provide interactive visualization of data collected by a Human BioMolecular Atlas Program (HuBMAP) (Jain et al., 2023) pancreas Tissue Mapping Center to facilitate exploration of the datasets via web-browser. We realized that other research teams have collected or will collect similar datasets on other types of tissue, and may also want to provide interactive visualizations of their own datasets. While there do exist large, centralized websites that host biology datasets and provide users with interactive data analysis capabilities (Börner et al., 2022), these other services do not provide researchers wide control over how the data is displayed. Our software gives researchers display interfaces out of the box, but also provides a model for how researchers may create other visualizations as they see fit for their own needs. While we developed this software for a future publication of pancreas data, we present here the Tissue Data Explorer along with provided synthetic sample data.

## Main Features

The software package consists of:

- a configuration app, which requires authentication and allows deployers to upload the data that will be displayed on the website
- a display app, which provides an interactive display for the data and allows viewers to download the source data files

### Volumetric Map

The display app includes the ability to visualize molecular spatial data collected throughout a 3D volume grid. The tool provides four different visualization modes for the volume grid so that researchers can easily visualize the magnitude of the molecular measurements taken across the tissue sample, including interior measurements. The volumetric map can display multiple different measurements, and can filter the tissue sample by layer or by inclusion in a category. Users can also adjust the color scheme and opacity of the visualizations.

### 3D Models

The display app can display 3D models of a tissue sample or organ. Multiple 3D models can be loaded together to display a set of regions. When a user hovers over an object, the application displays the coordinates of the point and the name of the object. When a user clicks on an object that is identified as a sample block, the app displays additional information about the block in the gray panel on the right-hand side of the model.

### Image Stack Viewer

The display app also handles image stacks. Image stacks are loaded into the app as a series of .PNG files along with the source files. The .PNGs are used for the website display and the source files are made available for download. All image sets that are available for a tissue block are listed together, and users can click an image set to view and download the images. The .PNG images are displayed with a slider that allows users to scroll through the images.

### Report Links

If the researchers have additional reports, such as Bioconductor HTML-based reports (Huntley et al., 2013), that are hosted elsewhere, they can configure links to those reports within the app.

### Data Upload

The volumetric map data, 3D models, image stacks and source files, and report links can all be uploaded to the display site using the configuration site. The configuration site validates the uploaded data before moving it into a shared location for the display site to access. The configuration site also provides downloads for example files that describe the required formats for the data.

## Technical Design

The project is containerized using Docker (Docker, 2024), which facilitates website setup because users can run a copy of the website without manually installing and managing the software dependencies (Ferlanti et al., 2023). The display site and configuration site are deployed with separate Docker containers that both point to a shared Docker volume, where the configuration site publishes validated data for display by the display site. The two sites are independent, so the configuration site can be taken down once the researchers have completed loading their data.

If the researchers are satisfied with the existing interfaces, they can deploy a functional website without writing any new code. Both sites are written in Python with Plotly Dash, which provides a Flask server and Python wrappers for React components. This allows developers to build interactive data visualizations without writing any JavaScript, so if researchers want to add new interfaces to their version of the site they can do so in Python.

## Figures and Tables

**Figure 1:**
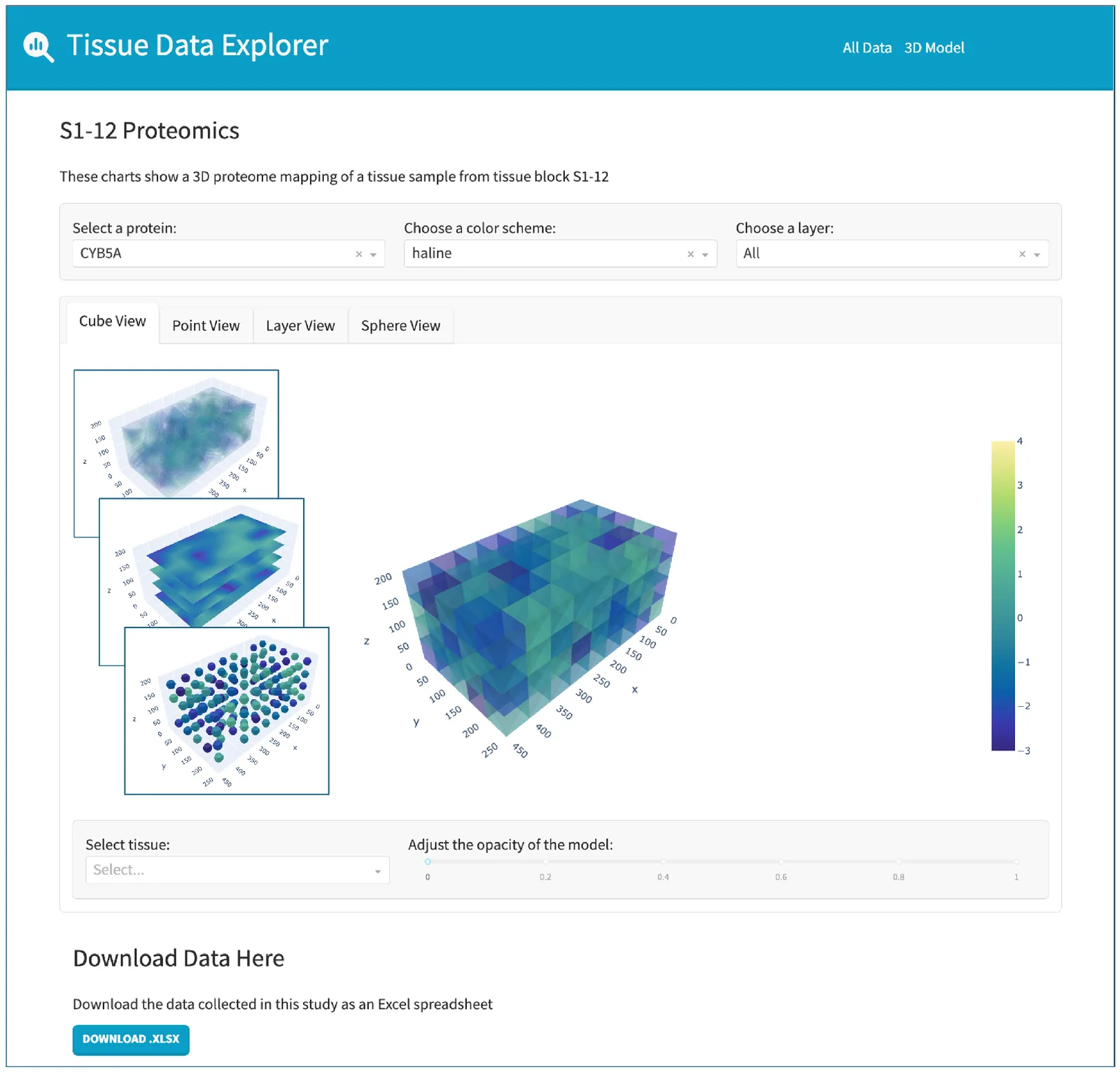
Visualizing synthetic data using the volumetric map interface. Insets show different selectable visualization modes.

**Figure 2:**
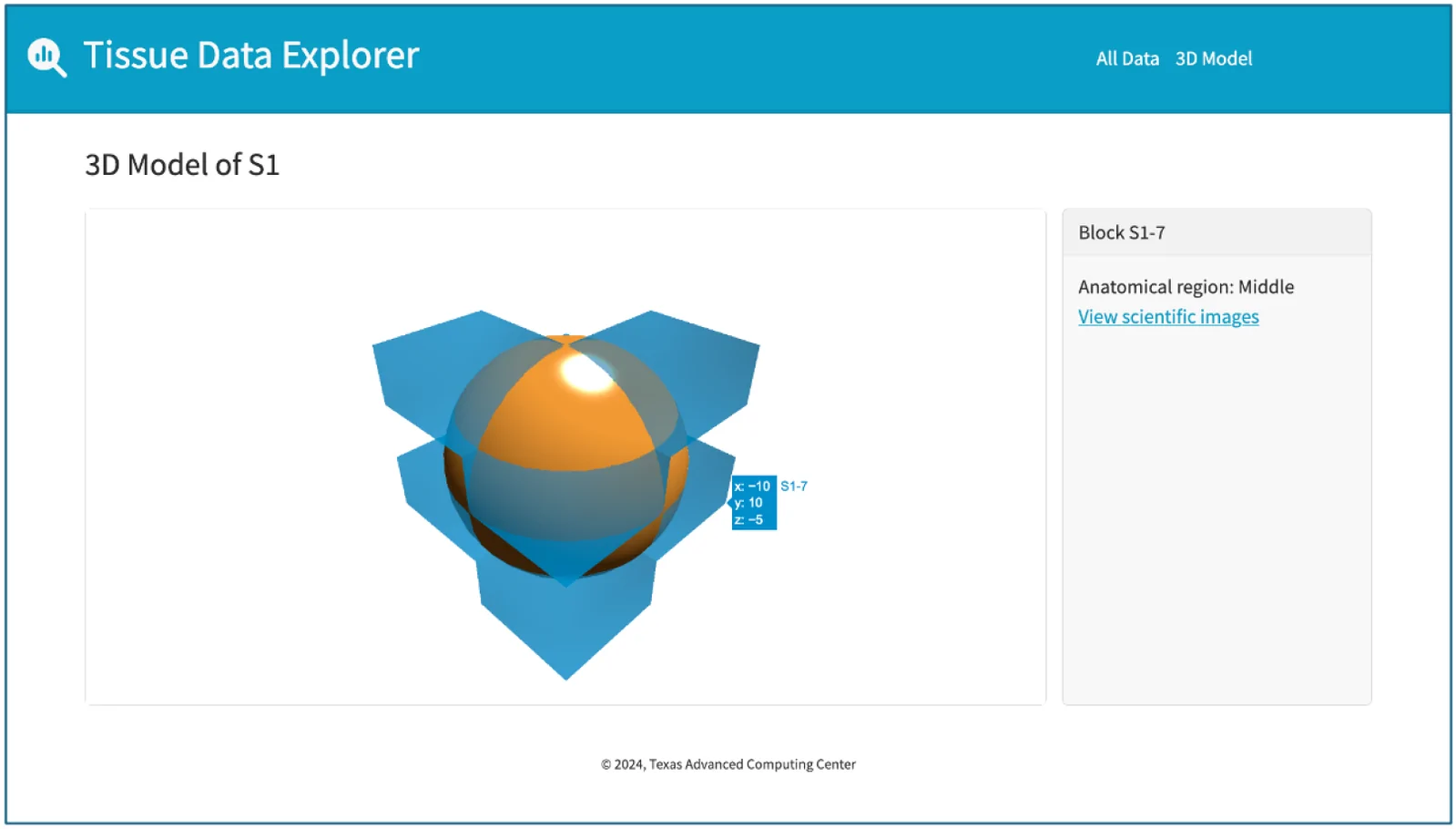
An interactive 3D model of an example synthetic tissue structure (orange sphere) with sample tissue blocks shown in blue. When selecting a specific block, the link to the data collected from that block is provided.

**Figure 3:**
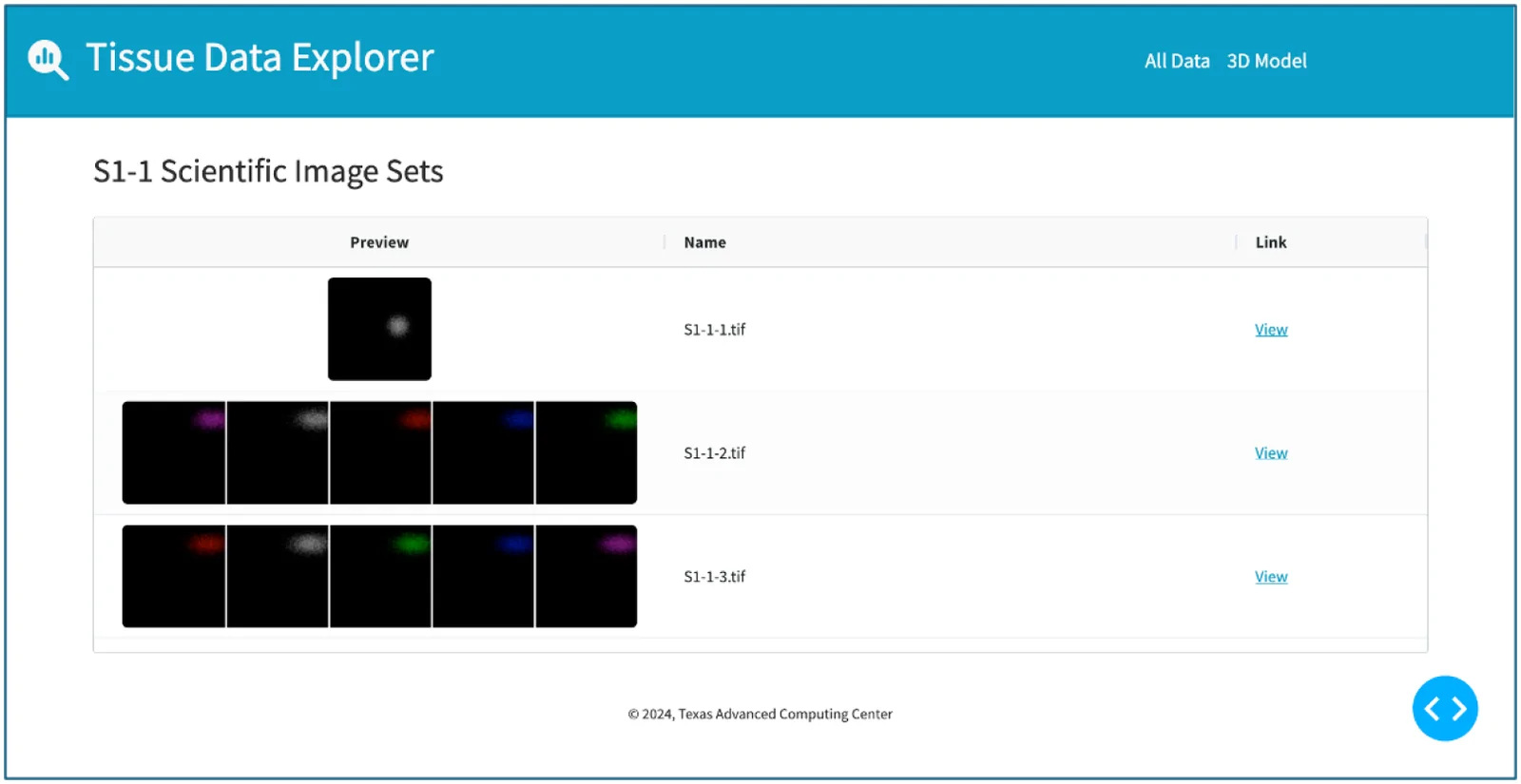
Image sets associated with a tissue block.

**Figure 4:**
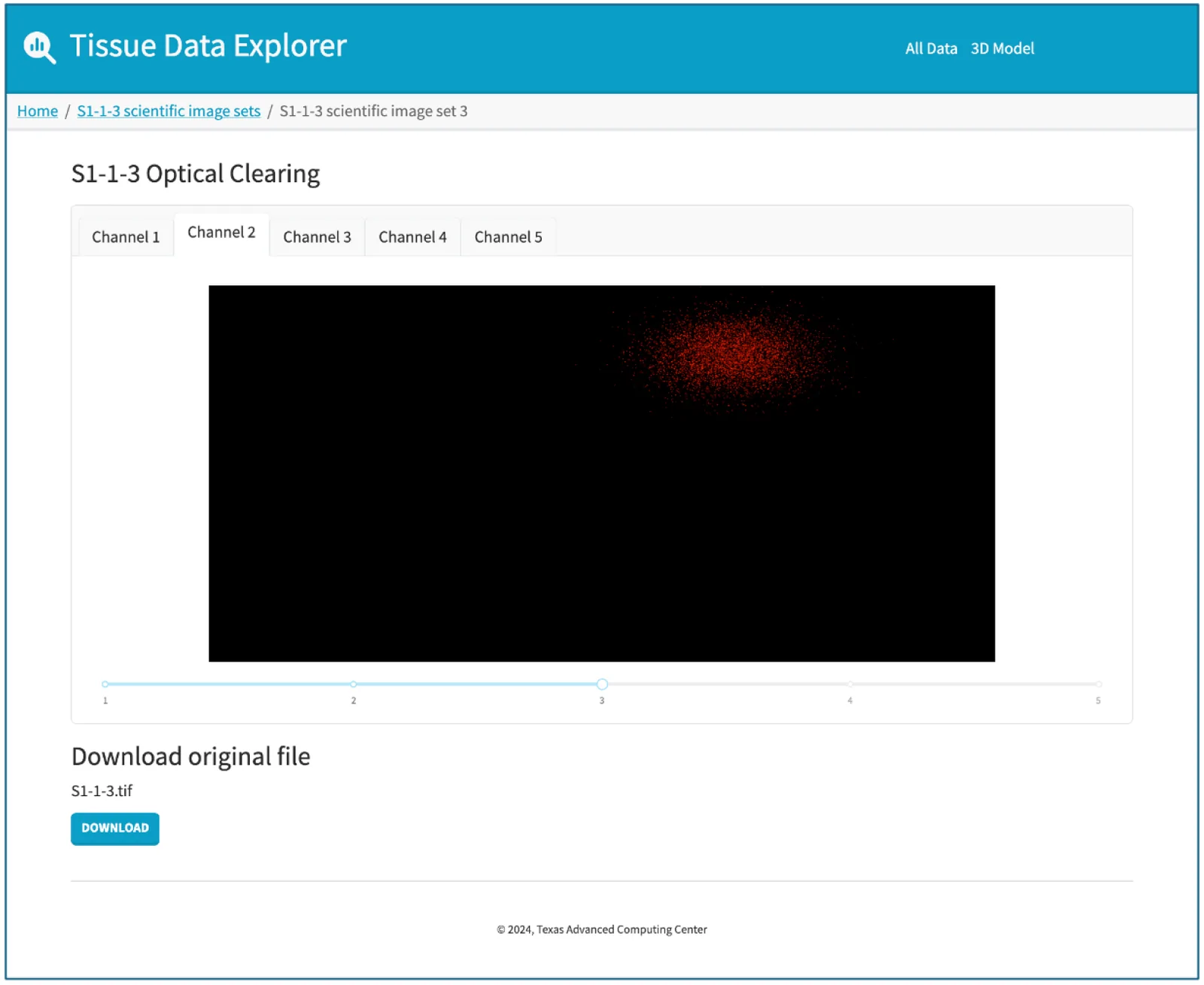
Interface for exploring a multichannel stack of images, shown here with a synthetic image stack.

**Figure 5:**
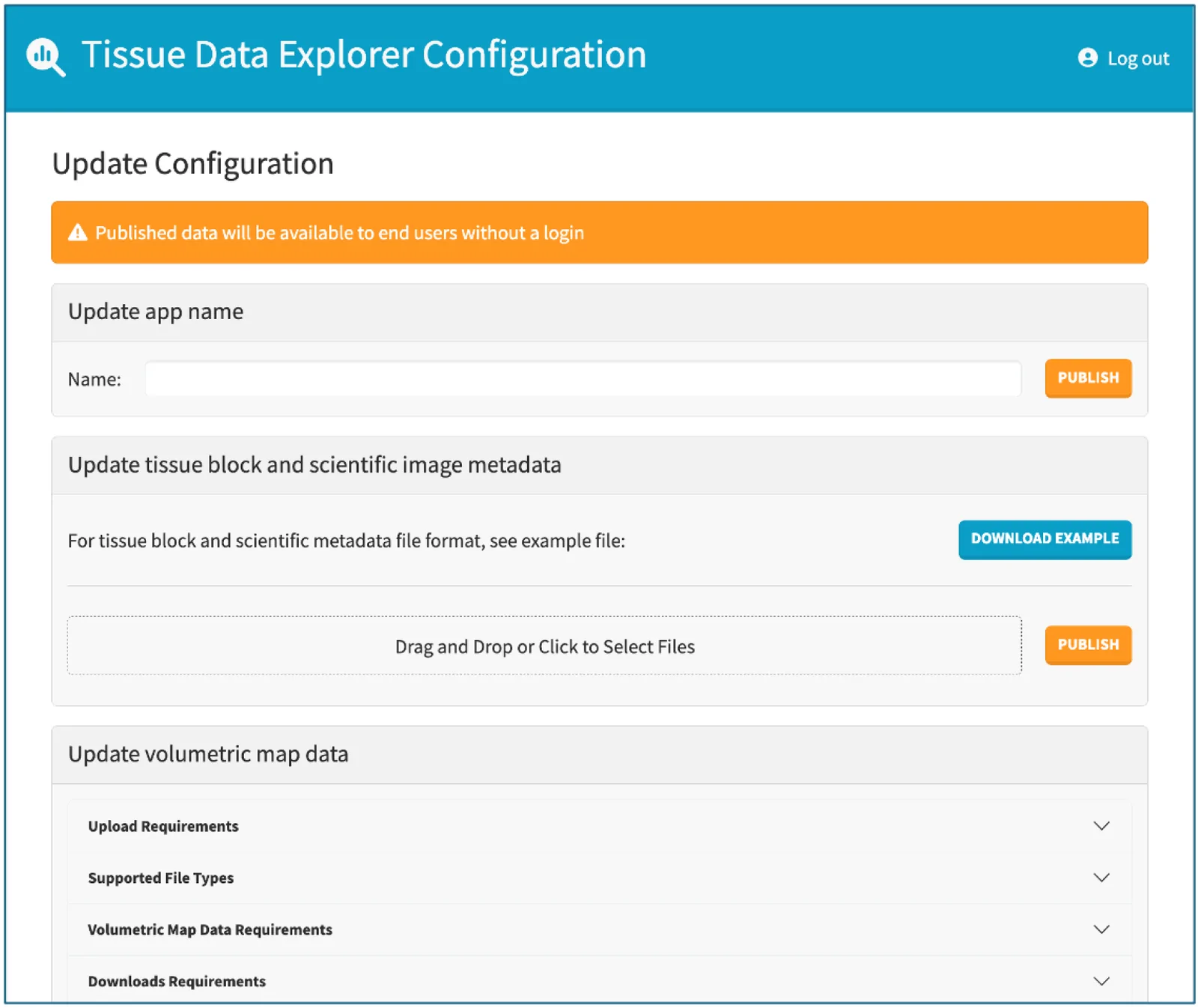
The configuration portal.

## Data Availability

Review Repository Archive
